# Aphasia following tibial fracture surgery: A case report

**DOI:** 10.1097/MD.0000000000044566

**Published:** 2025-10-03

**Authors:** Fang Wu, Yifan Zhang, Xu Zhu, Xian Zhang, Congye Li, Aizhong Wang

**Affiliations:** a Department of Intensive Care Medicine, Shanghai Jiao Tong University School of Medicine Affiliated Sixth People’s Hospital, Shanghai, China; b Department of Anesthesiology, Shanghai Jiao Tong University School of Medicine Affiliated Sixth People’s Hospital, Shanghai, China.

**Keywords:** aphasia, case report, fat embolism, magnetic resonance imaging, tibial fracture

## Abstract

**Rationale:**

Fat embolism syndrome is a serious and potentially life-threatening complication associated with long bone fractures and orthopedic procedures. Cerebral fat embolism (CFE), a rare neurological manifestation of fat embolism syndrome, typically manifests with nonspecific encephalopathic symptoms. Isolated focal neurological deficits, such as aphasia, are highly uncommon and seldom reported. This case illustrates an atypical presentation of CFE characterized by acute, isolated aphasia to enhance clinical awareness and prompt recognition.

**Patient concerns:**

A 57-year-old male developed acute expressive aphasia 1 hour following intramedullary nailing of a tibial fracture, in the absence of respiratory distress, hemodynamic instability, or dermatologic abnormalities.

**Diagnoses:**

Brain magnetic resonance imaging revealed the characteristic “starfield” pattern, confirming the diagnosis of CFE based on clinical history and laboratory findings.

**Interventions:**

Management included comprehensive supportive care: supplemental oxygen, intravenous glucocorticoids, and subcutaneous low-molecular-weight heparin.

**Outcomes:**

By the fourth postoperative day, his condition had stabilized. His language function improved gradually over the following days, and he was discharged 1 week after surgery, able to produce short sentences with occasional pauses.

**Lessons:**

This case highlights that CFE can present with isolated aphasia. Early magnetic resonance imaging utilizing diffusion-weighted imaging is essential to confirm the diagnosis. A high clinical suspicion for CFE must be maintained in any patient exhibiting acute neurological deficits following orthopedic surgery to ensure timely recognition and intervention.

## 1. Introduction

Fat embolic syndrome (FES) is a clinical syndrome caused by the entry of fat particles into the bloodstream, primarily due to fractures or orthopedic surgeries in the lower extremities, especially the femur.^[1]^ Other causes, such as bone marrow transplant and liposuction can also lead to FES, resulting in multi-system damage involving the lungs, brain, skin and other organs.^[2–4]^ The exact pathogenesis of FES remains unclear, but it is generally attributed to microvascular occlusion by fat emboli, local inflammatory responses, and endothelial injury. The classic triad of FES includes respiratory distress, neurological impairment, and petechial rash. Cerebral fat embolism (CFE), a rare yet potentially fatal manifestation, typically presents with acute disturbances in consciousness, seizures, or other neurological deficits.^[5–8]^ While an acute confusional state is more common, focal neurological signs are rare.^[2,3,5,6,9]^ Cases of CFE with aphasia as the primary symptom are exceptionally rare, with limited literature and no established diagnostic or treatment guidelines. This article presents a case of CFE characterized by prominent aphasia, aiming to explore its pathogenesis, diagnostic challenges, and therapeutic strategies to enhance clinical awareness and practice.

## 2. Case presentation

A 57-year-old male, 172 cm in height and weighing 70 kg, sustained left tibia and fibula fractures in a motorbike accident. He was classified as ASA Physical Status Class 1 (healthy patient) with no significant medical history or abnormal preoperative findings. Following the injury, low-molecular-weight heparin (4100 IU) was administered subcutaneously daily to prevent thromboembolism. Three days later, he underwent open reduction and internal fixation with intramedullary nailing of the left tibia. Ultrasound-guided femoral and sciatic nerve blocks were performed using 0.2% ropivacaine, with 20 mL administered to each nerve. General anesthesia was induced with 200 mg of propofol and 10 µg of sufentanil. A size 4 laryngeal mask airway (LMA Supreme) was inserted, and mechanical ventilation was initiated. Anesthesia was maintained with a continuous infusion of 20 mg/h propofol and inhalation of 2% sevoflurane. The surgery lasted 1 hour and 25 minutes, with stable hemodynamics throughout.

**Figure 1.**
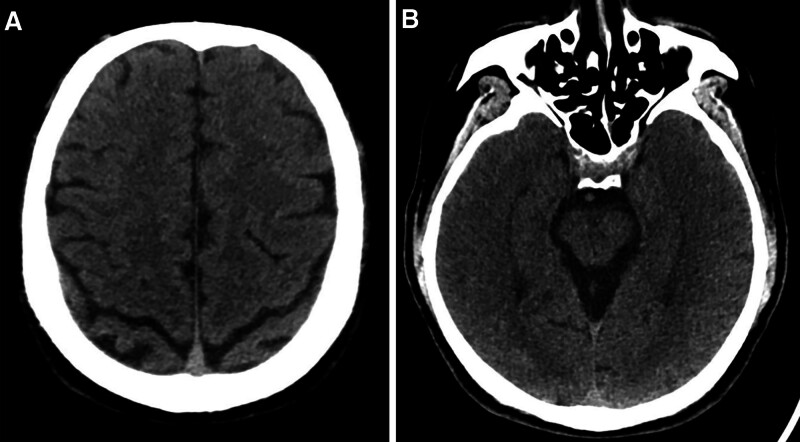
Head computed tomography performed 3 hours after the onset of aphasia reveals no significant hemorrhage or infarction. (A) Axial noncontrast image at the level of the frontal lobes shows normal parenchymal attenuation and gray–white matter differentiation. (B) Axial noncontrast image at the level of the occipital lobe demonstrates preserved architecture with no evidence of acute ischemia or hemorrhage. CT = computer tomograph.

Postoperatively, the patient presented with isolated aphasia with no other disturbances in consciousness. The glasgow coma scale score was E4V1M6. An ENT specialist was immediately consulted for laryngoscopic evaluation, which

preliminarily ruled out otolaryngological conditions, including arytenoid dislocation, as potential causes of aphasia. After 30 minutes, the aphasia improved slightly, and he was able to speak, although his speech remained slurred and indistinct. Head CT and CT angiogram performed 3 hours later showed no intracranial abnormalities (Fig. 1).

The following day, the patient was transferred to the intensive care unit, Upon admission, he exhibited an axillary temperature of 36.6°C, a heart rate of 109 beats per minute, blood pressure of 130/84 mm Hg, and oxygen saturation of 100%. He was conscious but spoke intermittently with difficulty (GCS E4V2M6). Pupils were equal, round, and 3 mm in diameter, with intact light reflexes. No visual field defects, blurred vision, or diplopia were noted. Muscle strength and tone were normal in all limbs, with absent bilateral Babinski reflexes. Petechial rashes were observed on the eyelids or chest wall.

**Figure 2.**
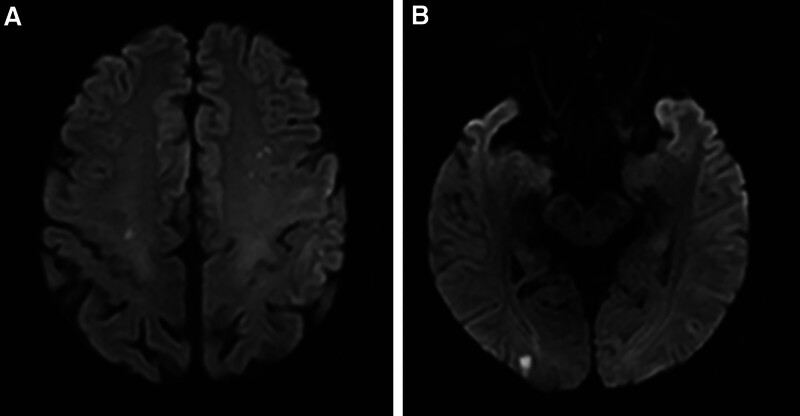
Head MRI of a patient with fat embolism syndrome shows scattered spots and patchy lesions with high-signal intensity on DWI in the bilateral frontal lobes (A) and right occipital lobe (B). DWI = diffusion weighted imaging, MRI = magnetic resonance imaging.

Laboratory results showed elevated D-dimer (19 mg/L) and CRP (44.1 mg/L), with a markedly elevated ESR of 120 mm/s. Postoperative hemoglobin levels were slightly reduced compared to preoperative levels and the platelet level was mildly decreased. Arterial blood gas analysis indicated a pH of 7.50, PCO₂ of 33.6 mm Hg, and PO₂ of 104 mm Hg. Qualitative chyle tests in blood and urine were negative. Echocardiography revealed normal atrial and ventricular sizes, with no segmental wall motion abnormalities or right-to-left shunt. Chest X-ray was unremarkable. Due to unexplained sudden onset of aphasia, a brain magnetic resonance imaging (MRI) was promptly conducted. The results revealed scattered spots and patchy high-signal shadows on DWI in the bilateral frontal lobes and the right occipital lobe (Fig. 2). Combining the clinical history, laboratory findings, and MRI results, the patient was diagnosed with CFE. He received comprehensive treatment, including intravenous glucocorticoids for 3 consecutive days and low-molecular-weight heparin 4100 IU subcutaneously once daily. There were no adverse and unanticipated events during the treatment. By the fourth postoperative day, his condition had stabilized, allowing for transfer out of the intensive care unit. Over the following days, his language function improved, and he was discharged 1 week after surgery, able to speak short sentences with brief pauses.

## 3. Discussion

In this case report, we present a rare case of CFE presenting with isolated aphasis as the initial symptom after tibial fracture surgery. Gurd and Wilson proposed the widely accepted clinical guidelines for diagnosing FES in 1974 (Table 1).^[10]^ The key manifestations of FES include pulmonary compromise, petechial hemorrhage, and neurological symptoms, typically occurring 12 to 36 hours after injury.^[2–4,11]^ When emboli reach the brain, they can result in CFE. The incidence of CFE may reach up to 15% following orthopedic surgeries, varying depending on the surgical sites.^[6]^

**Table 1:** Proposed diagnostic criteria of FES by Gurd and Wilson.

Criterion	Features
Gurd and Wilson criteria (1major + 4 minor)	Major criteria
	Respiratory insufficiency
	Cerebral involvement
	Petechial rash
	Minor criteria
	Tachycardia
	Fever
	Retinal changes
	Jaundice
	Renal changes (anuria or oliguria)
	Elevated ESR
	Thrombocytopenia
	Fat macroglobulinemia

FES = fat embolism syndrome.

Patients with CFE commonly present with impaired consciousness, altered mental status, or focal neurological deficits. In this case, the patient exhibited aphasia as the primary symptom following orthopedic surgery, with an acute onset characteristic of CFE. Approximately 30 minutes later, he could produce vocal sounds but was unable to speak coherently, despite remaining conscious throughout. Brain MRI revealed scattered spots and patchy leisions with high signal intensity on DWI in the bilateral frontal lobes and right occipital lobe, consistent with the radiological features of CFE.

The aphasia in this case likely resulted from the selective embolization of fat particles into the middle cerebral artery branches supplying the frontal and temporal lobes, leading to transient ischemia in language-critical areas. The MRI findings of scattered lesions in the bilateral frontal lobes and right occipital lobe support this mechanism. The patient’s partial recovery aligns with the transient nature of the ischemic insult caused by fat emboli, further reinforcing the diagnosis of FES. While CFE typically presents with multifocal neurological deficits, cases with isolated aphasia as the predominant symptom are exceedingly rare. The underlying mechanism may involve fat emboli selectively affecting cerebral vasculature in the dominant language areas. This case provides direct evidence of fat emboli specifically targeting the language centers, highlighting the unique pathophysiology of CFE.

## 4. Pathogenesis

The pathogenesis of CFE is primarily explained by 2 hypotheses: the mechanical obstruction and biochemical mechanisms.

**Mechanical obstruction hypothesis:** Fat emboli obstruct the microcirculation in brain tissue during orthopedic surgery, particularly during marrow expansion and intramedullary instrumentation. As intramedullary pressure increases, fat particles can enter the venous system.^[12]^ Duwelius et al confirmed this mechanism using ultrasound, demonstrating fat emboli entering the bloodstream during intramedullary cavity manipulation.^[13]^ Although pulmonary arterioles have a diameter of approximately 10 micrometers, fat emboli, being more deformable than blood clots, can traverse pulmonary capillary beds, leading to systemic embolism, including brain involvement. This may explain why most CFE cases present as scattered, punctate high-density signals without prominent focal signs. Another perspective proposes that emboli bypass the pulmonary circulation via a right -to-left shunt, such as a patent foramen ovale, or through transpulmonary passage.^[14]^

**Biochemical hypothesis:** The breakdown of fat releases free fatty acids and inflammatory mediators, damaging the blood-brain barrier and trigger inflammatory responses in brain tissue. In this case, the patient experienced severe neurological symptoms without pulmonary manifestations, consistent with previous reports.^[15,16]^ The immediate onset of aphasia following intramedullary reaming strongly supports a mechanical obstruction mechanism. Cardiac ultrasound excluded the presence of a patent foramen ovale, suggesting that the highly selective damage to the language centers may result from the preferential entry of fat emboli into branches of the middle cerebral artery.

Other mechanisms, such as the re-agglutination theory, may also contribute, but further research is needed to validate this hypothesis.

## 5. Diagnostic challenges

The definitive diagnosis of CFE relies on a comprehensive clinical assessment, incorporating medical history, imaging findings, and laboratory tests. Brain MRI is most reliable and sensitive tool for diagnosing CFE, typically revealing multiple high-signal dots on T2-weighted imaging, forming the characteristic “starfield” pattern.^[17]^ Suzuki proposed that this pattern, observed in the border zones of the vascular regions, indicates capillary obstruction caused by fat particles.^[18]^ Recent studies increasingly utilize MRI to confirm or rule out CFE.^[19]^

In this case, a head CT and vascular CTA performed on the night of symptom onset were negative. However, brain MRI conducted the following day revealed scattered spots and patchy areas of high-signal intensity on DWI in the bilateral frontal lobes and the right occipital lobe, consistent with CFE. These findings likely explain the patient’s aphasia, as the frontal lobes are crucial for language production and executive functions, while the occipital lobe is primarily responsible for visual processing.

Laboratory findings associated with FES often include decreased hemoglobin and platelet levels, elevated CRP, ESR, and FDPs, as well as prolonged prothrombin and thrombin times. The detection of fat particles in the blood, sputum, or urine can support the diagnosing of FES, and patients frequently present with hypoxemia. A positive chyluria test in blood or urine may also serve as a diagnostic indicator.

In this case, laboratory findings revealed mild decreases in hemoglobin and platelet levels, elevated FDPs and ESR, and prolonged thrombin and prothrombin times. However, qualitative chyle tests in blood and urine were negative. It is important to note that the presence of fat particles or a positive chyluria test does not definitively confirm FES,^[20,21]^ as their specificity ranges from 67% to 95%.^[7,22]^ Therefore, the diagnosis of FES requires comprehensive clinical evaluation.

## 6. Treatment and prognosis

The treatment of CFE is primarily supportive, including aggressive respiratory support, circulatory stabilization, and early rehabilitation interventions. In this case, comprehensive treatment led to significant improvement in the patient’s aphasia, underscoring the critical role of early recognition and intervention in improving outcomes. 0 Literature suggests that most patients with CFE have a good prognosis, although severe cases may result in permanent neurological deficits.

## 7. Clinical significance and implications

This case of CFE presenting as isolated aphasia highlights the critical importance of thorough history-taking and multidisciplinary collaboration in suspected cases. In patients with a clear history of trauma or intramedullary nailing procedures, nonspecific central nervous system symptoms should raise a high index of suspicion for CFE. Additionally, the integration of advanced imaging techniques with meticulous clinical observation can significantly enhance early diagnostic and therapeutic strategies for CFE.

## 8. Conclusion

This case highlights a rare presentation of CFE with isolated aphasia, emphasizing the need for high clinical suspicion in patients with orthopedic surgery history, particularly in cases involving reaming procedures. Early brain MRI, identifying starfield-like hyperintense lesions, is crucial for diagnosis. Multidisciplinary collaboration improves outcomes, and further research into the pathogenesis and treatment of CFE is essential to enhance management and prognosis.

## Acknowledgments

The authors would like to thank the patient for agreeing to the publication of the article.

## Author contributions

**Conceptualization:** Fang Wu.

**Supervision:** Aizhong Wang.

**Writing – original draft:** Xu Zhu, Xian Zhang, Congye Li.

**Writing – review & editing:** Yifan Zhang, Xian Zhang, Aizhong Wang.
